# Supplementary material for: Anthropogenic stressors impact fish sensory development and survival via thyroid disruption
Source: Nat Commun. 2020 Jul 17;11:3614. doi: 10.1038/s41467-020-17450-8 (PMC7367887; doi:10.1038/s41467-020-17450-8)
Supplement: Supplementary file 1 — Supplementary Information [file 41467_2020_17450_MOESM1_ESM.pdf]

Supplementary Information for

Anthropogenic stressors impact fish sensory development and survival via thyroid disruption

Besson et al.

This file includes:

Supplementary Figures 1 to 14

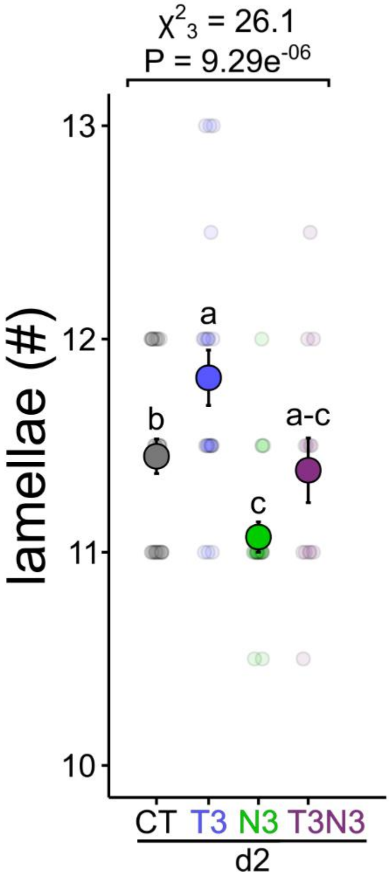

Supplementary Fig. 1. Variation in lamellae number across pharmacological treatments in d2 *Acanthurus triostegus*.

CT = control; T3 = T3-treatment; N3 = N3-treatment; T3N3 = combined T3-N3-treatment. Data are indicated as mean (opaque circles) ± SE (error bars), and transparent circles indicate each data point ($n = 86$). Letters indicate statistically different groups according to two sided Tukey posthoc-tests following COM-Poisson GLM (χ^2). Source data are provided as a Source Data file.

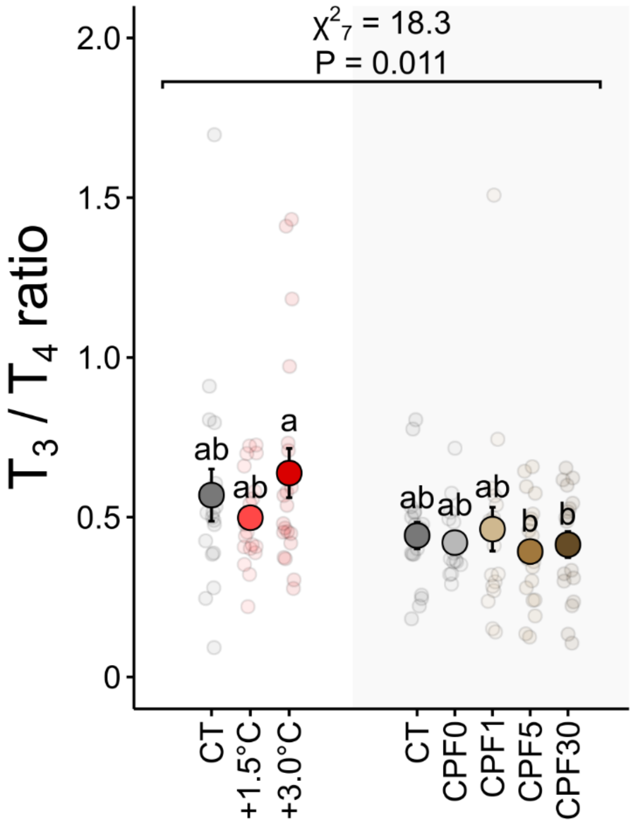

Supplementary Fig. 2. Impacts of increased temperature and chlorpyrifos (CPF) exposure on T_3/T_4 ratios.

Data are indicated as mean (opaque circles) ± SE (error bars), and transparent circles indicate each data point (n = 149). Letters indicate statistically different groups according to two sided Tukey posthoc-tests following Gamma GLM (χ^2). Source data are provided as a Source Data file.

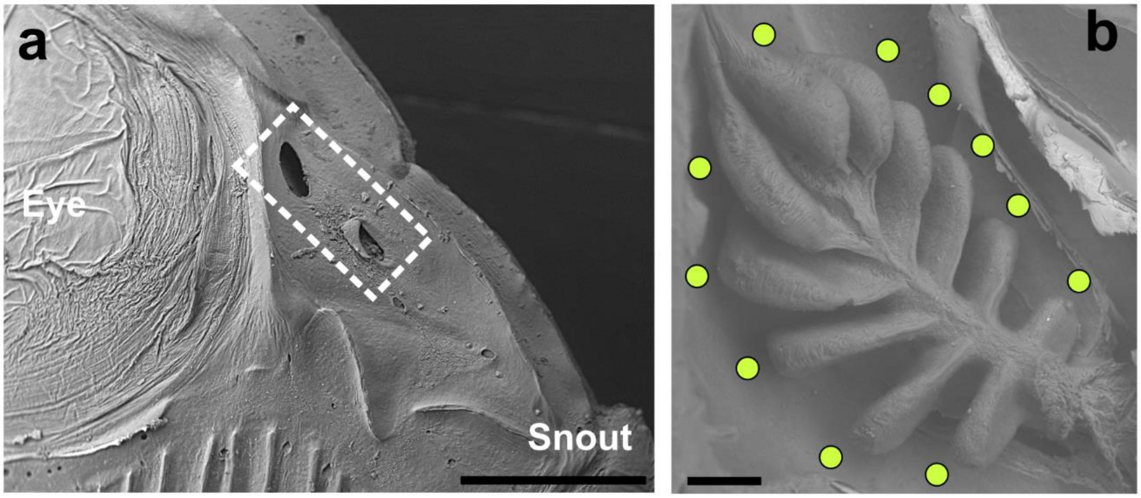

Supplementary Fig. 3. Olfactory organ location and morphology in a d0 *Acanthurus triostegus*.

a Scanning electron microscope (SEM) photograph of *A. triostegus* head (right side of the head) localized around the right olfactory organ (white dotted-rectangle) between the eye and the snout. Dotted area indicates the region that is dissected (removal of the skin layer between the two nares) and presented in b. Scale bar indicates 1 mm. **b** SEM photograph of a rosette with 11 lamellae (light green dots). Scale bar indicates 100 µm.

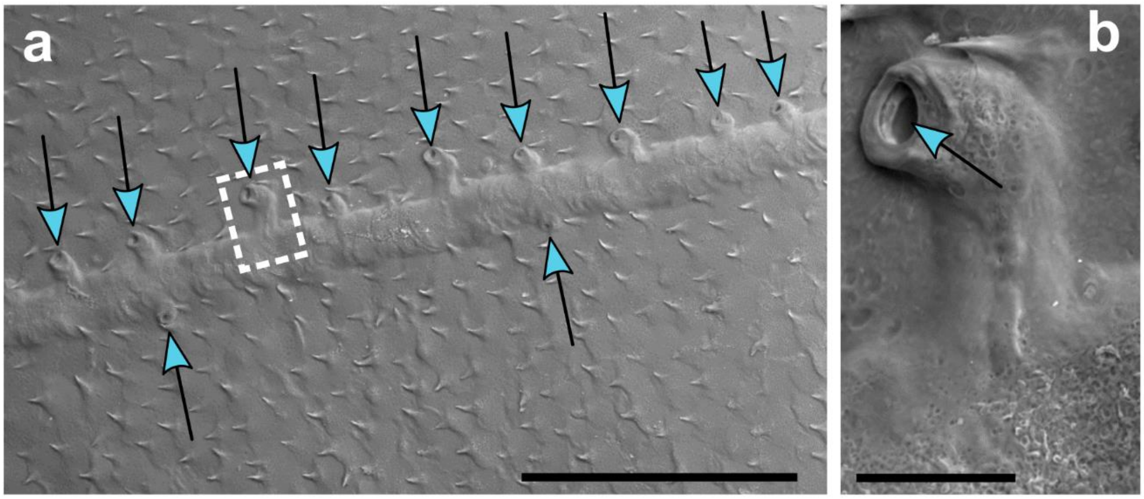

Supplementary Fig. 4. Location and morphology of trunk canal pores in a d0 *Acanthurus triostegus*.

a Scanning electron microscope (SEM) photograph of the right body flank where the trunk canal and its pores are visible (light blue arrows). The dotted white rectangle represents the magnified region shown in B. Scale bar indicates 1 mm. **b** SEM photograph of a trunk canal pore (blue arrow). Scale bar indicates 100 µm.

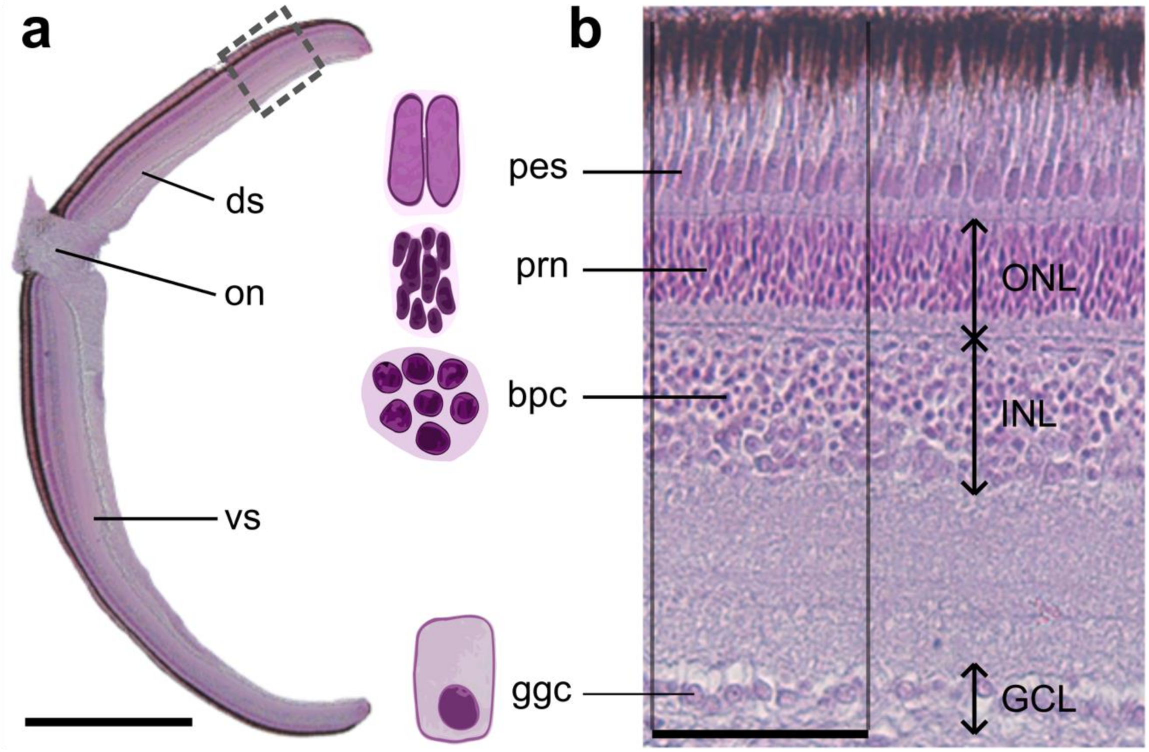

Supplementary Fig. 5. Cross-sections in the retina of a d0 *Acanthurus triostegus*.
a Cross section in the retina stained with hematoxylin/eosin, showing the dorsal side (ds), the ventral side (vs), and the optical nerve (on). The dotted gray rectangle represents the magnified region shown in B. Scale bar indicates 100 µm. **b** Cross section in the retina stained in hematoxylin/eosin, consisting of the ganglion cell layer (GCL), inner nuclear layer (INL) and outer nuclear layer (ONL) and containing the ganglion cells (ggc), bipolar cells (bpc) and photoreceptor nuclei (prn). In the anterior part of the retina (top of the picture), photoreceptor external segments (pes) are identified. The two opaque vertical bars identify a 50 µm wide area, as used for cell counts and layer thickness measurements. Scale bar indicates 50 µm. Schematic representation of the four cell types are presented to the left of their legends.

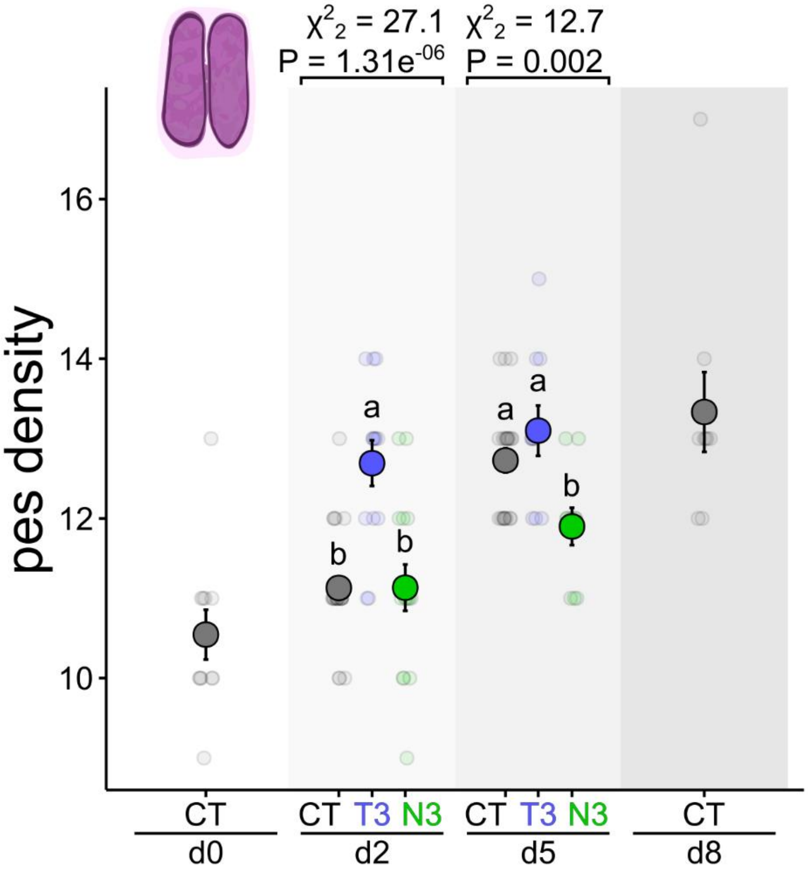

Supplementary Fig. 6. Densities of photoreceptor external segments (pes) in metamorphosing *Acanthurus triostegus*.

Changes in pes density between developmental stages (d0 to d8) and among treatments (CT = control; T3 = T3-treatment; N3 = N3-treatment). Data are indicated as mean (opaque circles) ± SE (error bars), and transparent circles indicate each data point (n = 113). Letters indicate statistically different groups according to two sided Tukey posthoc-tests following COM-Poisson GLM (χ^2). Source data are provided as a Source Data file.

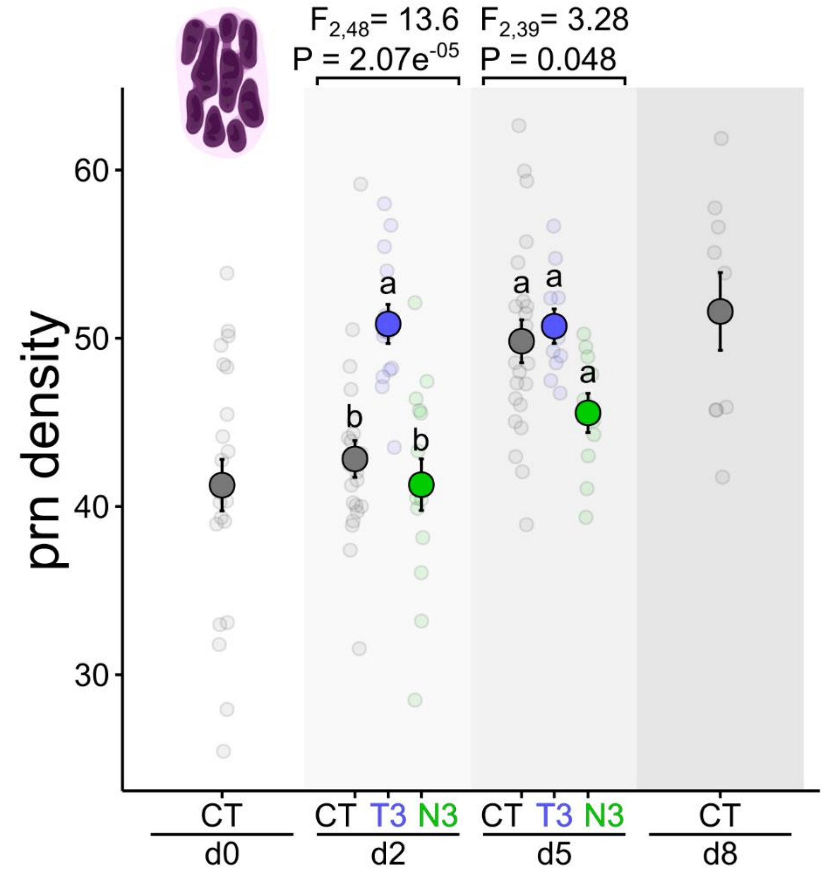

Supplementary Fig. 7. Densities of photoreceptor nuclei (prn) in metamorphosing *Acanthurus triostegus*.

Changes in prn density between developmental stages (d0 to d8) and among treatments (CT = control; T3 = T3-treatment; N3 = N3-treatment). Data are indicated as mean (opaque circles) ± SE (error bars), and transparent circles indicate each data point (n = 125). Letters indicate statistically different groups according to two sided Tukey posthoc-tests following LM (F). Source data are provided as a Source Data file.

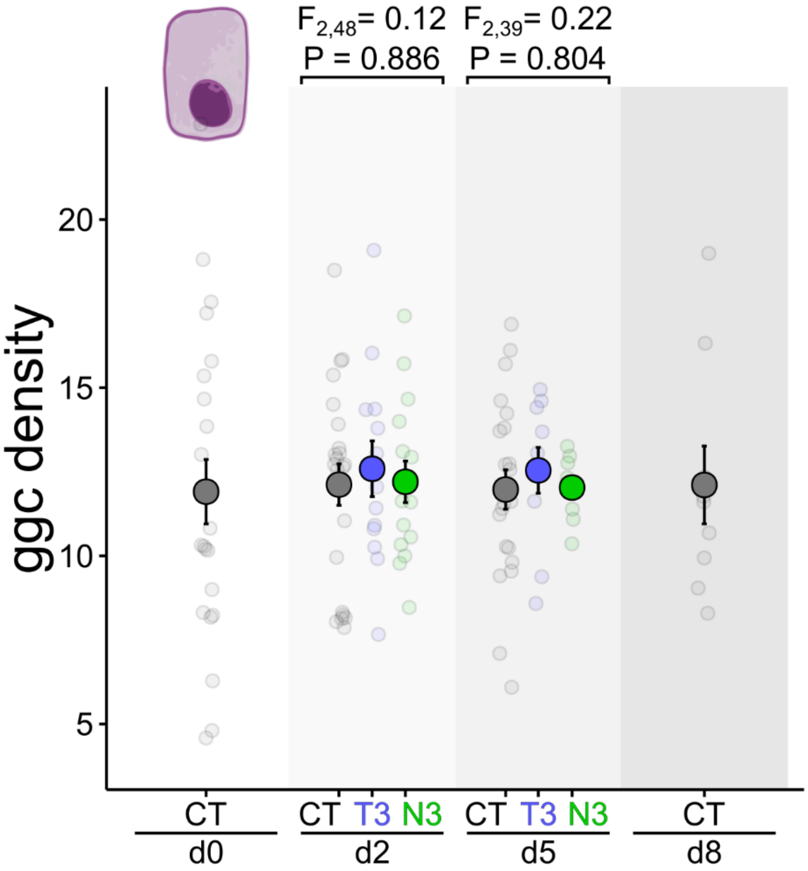

Supplementary Fig. 8. Densities of ganglion cells (ggc) in metamorphosing *Acanthurus triostegus*.

Changes in ggc density between developmental stages (d0 to d8) and among treatments (CT = control; T3 = T3-treatment; N3 = N3-treatment). Data are indicated as mean (opaque circles) ± SE (error bars), and transparent circles indicate each data point (n = 125). Letters indicate statistically different groups according to two sided Tukey posthoc-tests following LM (F). Source data are provided as a Source Data file.

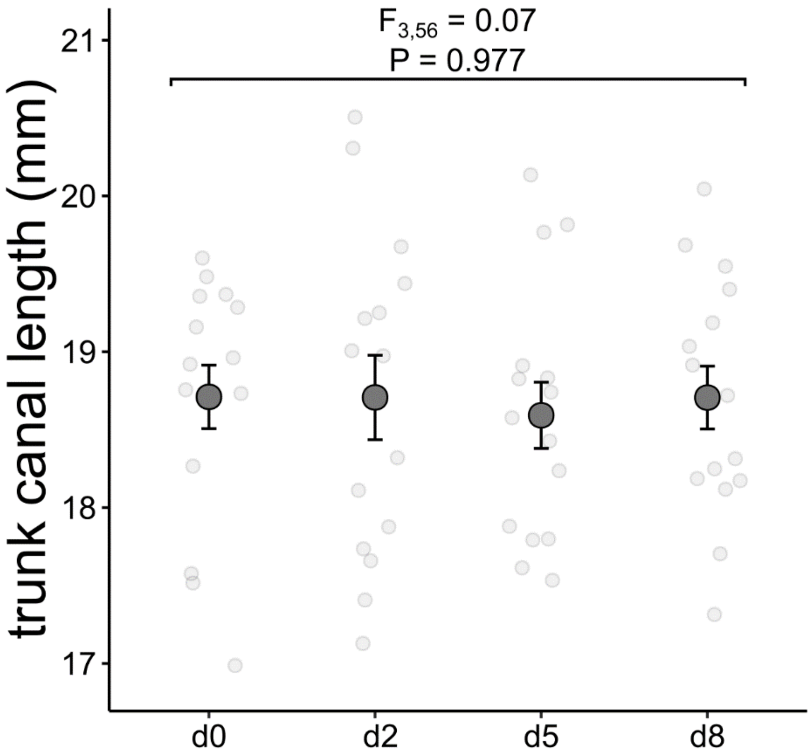

Supplementary Fig. 9. Variation in trunk canal length in metamorphosing *Acanthurus triostegus*.

Trunk canal length was assessed from day 0 (d0) to day 8 (d8). Data are indicated as mean (opaque circles) ± SE (error bars), and transparent circles indicate each data point (n = 60). Letters indicate statistically different groups according to two sided Tukey posthoc-tests following LM (F). Source data are provided as a Source Data file.

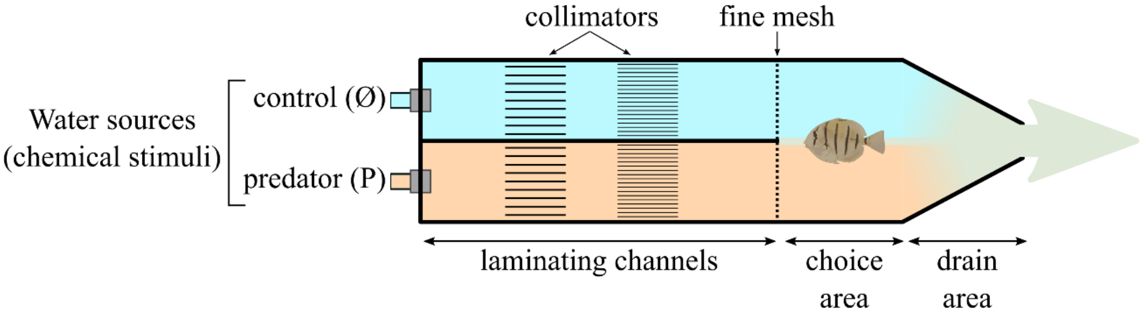

Supplementary Fig. 10. Design of the two-channel choice-flume apparatus.
In the two water inlets, seawater contained either no chemical cue (Ø, blue) or chemical cues from the predator *Lutjanus fulvus* (P, orange). Fish drawing represents a d2 *A. triostegus* (not to scale) in the choice area. Total length: 25 cm (laminating channel area: 15 cm; choice area: 7 cm and drain area: 5 cm); width: 5 cm; water height: 3 cm.

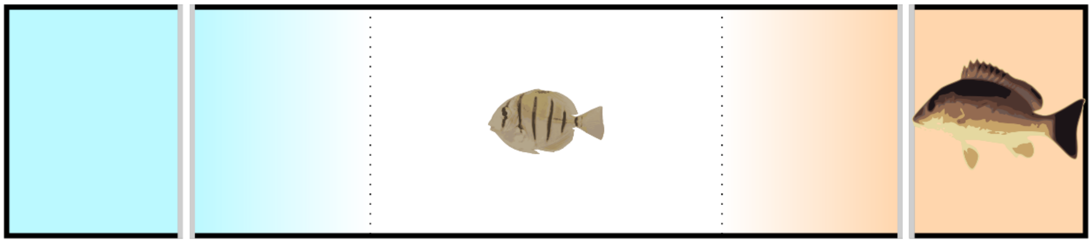

Supplementary Fig. 11. Design of the visual double-choice-tank apparatus.
At the two edges of the choice tank are two separate aquaria: an empty control tank (Ø, blue) and a tank with a live predator (*L. fulvus*) (P, orange), both containing an air stone (not represented here). Central fish represents a d2 *A. triostegus* (not to scale) in the no choice area. Dotted lines indicate the location of the removable opaque panels. Grey walls indicate the transparent walls of the aquaria, while black walls indicate the opaque walls. Total length: 60 cm (no choice area: 30 cm; each choice area: 15 cm); width: 15 cm; water height: 8 cm.

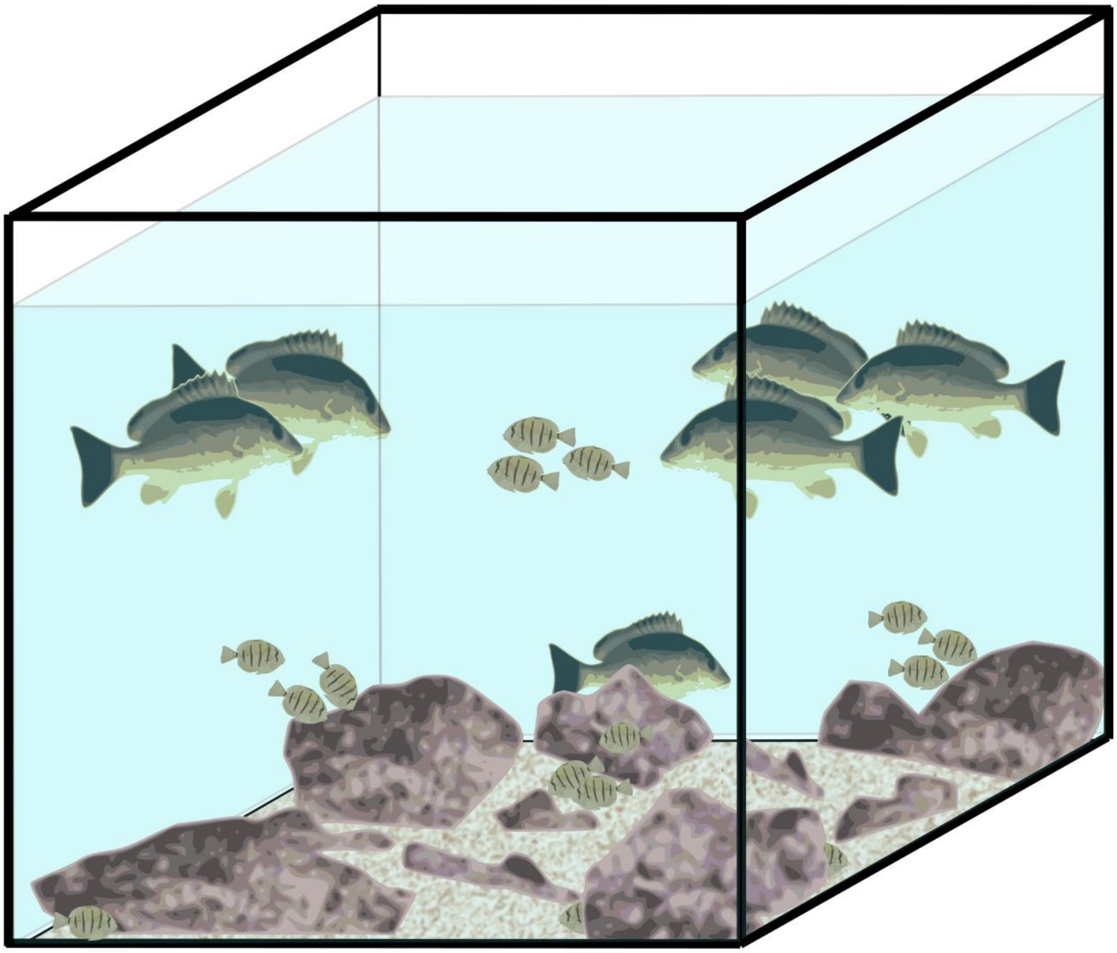

Supplementary Fig. 12. Design of the predation-arena.
Large fish represent predatory *L. fulvus*, while small fish represent *A. triostegus* that are tested for survival (not to scale). Each trial consisted of 6 *L. fulvus* and 45 *A. triostegus*. Bottom of the predation arena was covered with coral rubble and sand. Arena size: 1 x 1 x 1 m (L x W x H).

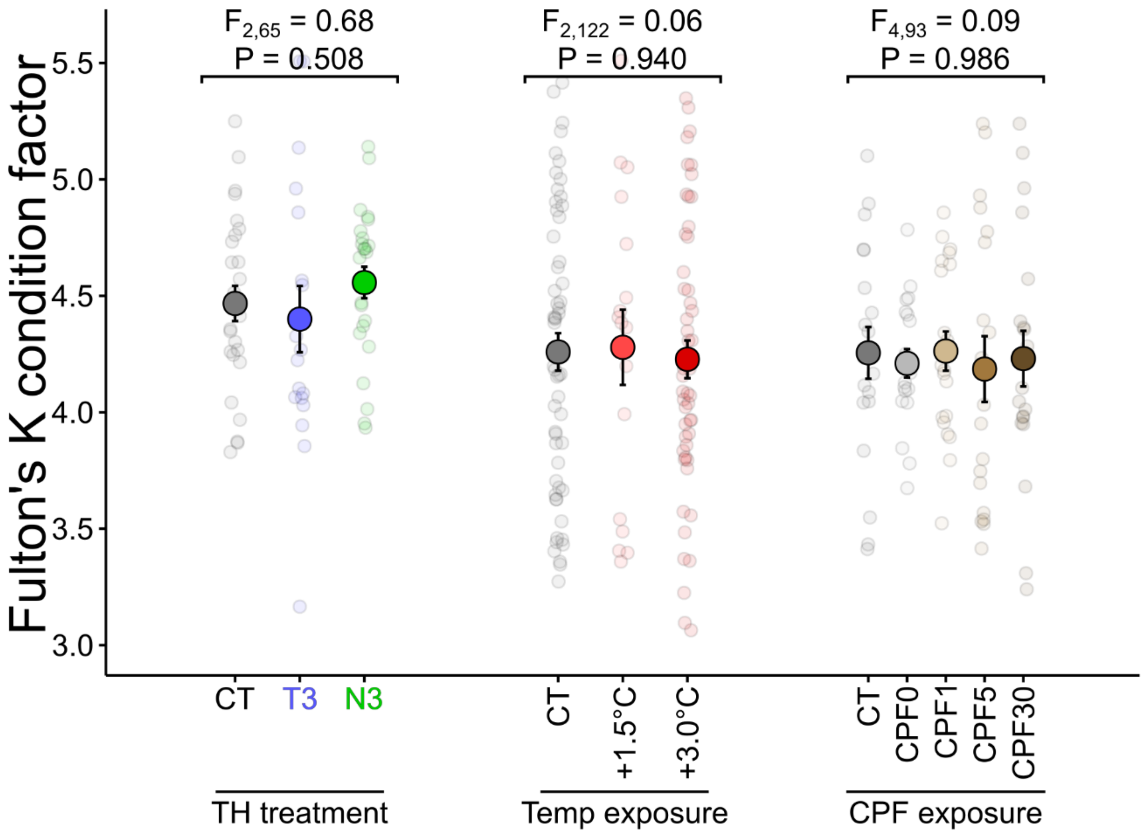

Supplementary Fig. 13. Variation in Fulton's K condition factor between treatments in d2 *Acanthurus triostegus*.

Fulton's K condition factor at d2 between thyroid hormones (TH), increased temperature (Temp), and chlorpyrifos (CPF) treatments. Data are indicated as mean (opaque circles) ± SE (error bars), and transparent circles indicate each data point (n = 303). Letters indicate statistically different groups according to two sided Tukey posthoc-tests following LM (F). Source data are provided as a Source Data file.

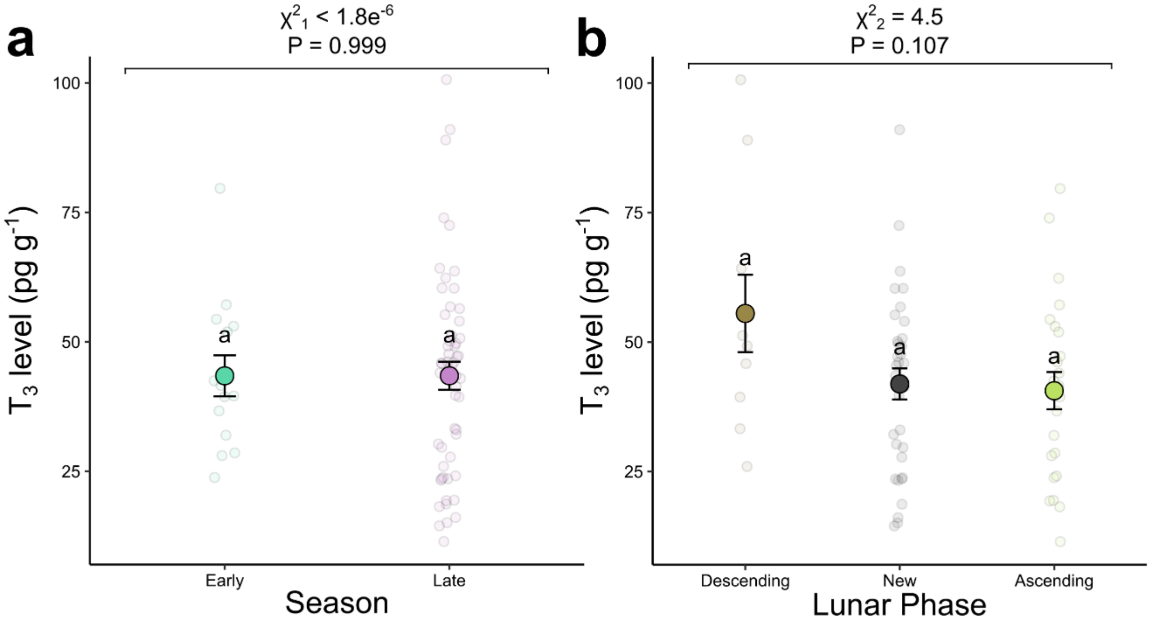

Supplementary Fig. 14. Variation in T3 levels across seasons (a) and lunar phases (b). Early indicates data from February 2015 while Late indicates data from September to November 2015. Descending indicates data from periods with moon visibility ranging from 75 to 25%, New indicates new moon period (i.e. visibility of the moon below 25%), and ascending indicates data from periods with moon visibility ranging from 25 to 75%. Data are indicated as mean (opaque circles) ± SE (error bars), and transparent circles indicate each data point (n = 68). P values and letters indicates outputs from two sided Tukey posthoc-tests following Gamma GLMEM. Source data are provided as a Source Data file.
