# Supplementary material for: Anthropogenic stressors impact fish sensory development and survival via thyroid disruption
Source: Nat Commun. 2020 Jul 17;11:3614. doi: 10.1038/s41467-020-17450-8 (PMC7367887; doi:10.1038/s41467-020-17450-8)
Supplement: Supplementary file 3 — Reporting Summary [file 41467_2020_17450_MOESM3_ESM.pdf]

nature research

Corresponding author(s): Marc Besson

Last updated by author(s): Jun 21, 2020

Reporting Summary

Nature Research wishes to improve the reproducibility of the work that we publish. This form provides structure for consistency and transparency in reporting. For further information on Nature Research policies, see Authors & Referees and the Editorial Policy Checklist .

Statistics

For all statistical analyses, confirm that the following items are present in the figure legend, table legend, main text, or Methods section.

n/a	Confirmed	
☐	☒	The exact sample size (*n*) for each experimental group/condition, given as a discrete number and unit of measurement
☐	☒	A statement on whether measurements were taken from distinct samples or whether the same sample was measured repeatedly
☐	☒	The statistical test(s) used AND whether they are one- or two-sided<br>*Only common tests should be described solely by name; describe more complex techniques in the Methods section.*
☐	☒	A description of all covariates tested
☐	☒	A description of any assumptions or corrections, such as tests of normality and adjustment for multiple comparisons
☐	☒	A full description of the statistical parameters including central tendency (e.g. means) or other basic estimates (e.g. regression coefficient) AND variation (e.g. standard deviation) or associated estimates of uncertainty (e.g. confidence intervals)
☐	☒	For null hypothesis testing, the test statistic (e.g. F, t, r) with confidence intervals, effect sizes, degrees of freedom and P value noted<br>*Give P values as exact values whenever suitable.*
☒	☐	For Bayesian analysis, information on the choice of priors and Markov chain Monte Carlo settings
☒	☐	For hierarchical and complex designs, identification of the appropriate level for tests and full reporting of outcomes
☒	☐	Estimates of effect sizes (e.g. Cohen's *d*, Pearson's *r*), indicating how they were calculated

Our web collection on statistics for biologists contains articles on many of the points above.

Software and code

Policy information about availability of computer code

Data collection	ImageJ v1.52a and RStudio v1.1.463 (R version 3.5.3) were used. R code is available at: https://doi.org/10.5281/zenodo.3742258
Data analysis	All statistical analyses were conducted using R version 3.5.3

For manuscripts utilizing custom algorithms or software that are central to the research but not yet described in published literature, software must be made available to editors/reviewers. We strongly encourage code deposition in a community repository (e.g. GitHub). See the Nature Research guidelines for submitting code & software for further information.

Data

Policy information about availability of data

All manuscripts must include a data availability statement. This statement should provide the following information, where applicable:

- Accession codes, unique identifiers, or web links for publicly available datasets
- A list of figures that have associated raw data
- A description of any restrictions on data availability

The source data underlying Figs 1e-g, 2a-c, 3a-f, 4a-b and Supplementary Figs 1, 2, 6-9, 13 and 14 are provided as a Source Data file. These data and their associated R-code (for statistical analyses and for figure construction) are also available at https://doi.org/10.5281/zenodo.3742258.

Field-specific reporting

Please select the one below that is the best fit for your research. If you are not sure, read the appropriate sections before making your selection.

☐ Life sciences ☐ Behavioural & social sciences ☒ Ecological, evolutionary & environmental sciences

Ecological, evolutionary & environmental sciences study design

All studies must disclose on these points even when the disclosure is negative.

Study description	This study investigates the ecological importance of TH function in recruiting and metamorphosing fish, as well as its sensitivity to two anthropogenic stressors (increased temperature and chlorpyrifos pesticide).<br><br>To test the role of TH on sensory organ maturation, behavior (response to sensory cues), and survival (predation test), the TH pathway in metamorphosing larvae was pharmacologically manipulated. A. triostegus were injected in their ventral cavity at d0 with 20 µl of a pharmacological treatment: (i) T3 + iopanoic acid (IOP) both at 10-6 M (T3-treatment), or (ii) NH3 at 10-6 M (N3-treatment). IOP was used as an inhibitor of deiodinase enzymes, following comparable work in mammals and amphibians, and as routinely used in fish to prevent the immediate degradation of injected T3. The T3-treatment was therefore applied to promote TH signaling. NH3 is a known antagonist of TH receptors (TR) in vertebrates and in A. triostegus in particular. NH3 prevents the binding of TH such as T3 to TR, therefore impairing the binding of transcriptional coactivators to TR, which therefore remain in an inactive and repressive conformation. The N3-treatment was thus applied to repress TH signaling by disrupting the TH pathway. T3 and NH3 were initially suspended in dimethyl sulfoxide (DMSO), at 10-2 M, and then diluted in Phosphate Buffered Saline (PBS) 1X to reach 10-6 M. Control treatment (CT) fish were injected with 20 µL of DMSO diluted 10.000 times in PBS 1X to control for the effect of the solvent and injection. DMSO and NH3 non-toxicity has been previously determined. Until subsequent sampling at d2 and d5, all fish were re-treated each morning (i.e. at d2, d3 and d4 for fish sampled at d5) to maintain pharmacological activity. Following collection and subsequent treatment, larvae were transferred to a nursery area on the north coast of the island (S17°29'26.5378", W149° 53'29.2252") where they were raised in in situ cages (cylindrical cages, diameter: 30 cm, height: 50 cm, 15 fish per cage). This allowed them to develop in in situ conditions. As A. triostegus feeds on algal turf following settlement, cages were stocked with a supply of turf-covered coral rubble that was replaced daily, ensuring both shelter and constant food availability. Fish were subsequently sampled on d2, d5, and d8, post-collection to examine sensory organ maturation, behavior (response to sensory cues), and survival (predation test).<br><br>To test the role of increased temperature and chlorpyrifos pesticide, following collection, d0 fish were maintained in groups of 10 individuals at the CRIOBE Marine Research Station. Each group was held in a 30Lx20Wx20H cm aquaria containing 12 L of filtered (1 µm filter) seawater. All tanks were subject to a 12:12 hour light-dark cycle (06:00 – 18:00 light period) and oxygenated with an air stone. For increased temperature treatments, seawater was in an open system, and water temperature was maintained at either 28.5°C, 30.0°C or 31.5°C. 28.5°C was chosen as the basal temperature as this was the mean temperature in the Moorea lagoon at the time of the study, and corresponds to the mean annual lagoon temperature in this region (http://observatoire.criobe.pf). Subsequent increases of +1.5°C and +3.0°C were selected as these are in line with end-of-century projections for tropical Pacific sea surface temperatures. Fresh coral rubbles were added to the tanks and replaced each day to provide shelter and food availability. For CPF exposure, five different treatments were applied: unaltered seawater (CT, control treatment), seawater with acetone at a final concentration of 1:1.000.000 (CPF0, solvent control treatment, as CPF was made soluble using acetone), or seawater with CPF at a nominal concentration of either 1, 5, or 30 µg L-1 (CPF1, CPF5, and CPF30 treatments) as similar to our past work. These concentrations were selected based on the findings of recent studies of coral-reef fishes exposed to CPF. Aquaria used for CPF exposure treatments and associated controls were not equipped with coral rubble to prevent potential interaction with the pesticide. Water was replaced each day to ensure the maintenance of CPF concentrations. For combined increased temperature and CPF exposure treatments, fish were also maintained without coral rubble with water replaced daily. For treatments where fish were exposed to an anthropogenic stressor and provided with supplemental-T3, fish were maintained in either the elevated temperature or CPF exposure treatments as described above but were also injected with either T3 (T3-treatment) or with DMSO (CT, to control for solvent and injection). These injections were done as described above (thyroid hormone signaling experiment: CT, T3- and N3-treatments).
Research sample	The convict surgeonfish Acanthurus triostegus (Linnaeus, 1758) is an abundant coral reef-associated species found throughout the Indo-Pacific, including around Moorea Island, French Polynesia. It has a pelagic larval duration of 53 ± 8 days after which larvae settle back to the reef. A. triostegus is a well-studied species with regards to metamorphosis, with a well-defined developmental sequence. The recruitment of A. triostegus larvae to reef-habitats coincides with a true metamorphosis into juveniles, with this full process taking around one week. Its metamorphosis is controlled by thyroid hormones (TH, the precursor thyroxine (T4), and the active hormone triiodothyronine (T3)), which is the same as other teleosts and other metamorphosing vertebrates such as amphibians. We recently showed that TH signaling in larval A. triostegus is vulnerable to disruption by anthropogenic stressors, including the waterborne pesticide chlorpyrifos and artificial light at night. Given the importance of TH during teleost metamorphosis, and as metamorphosis coincides with recruitment in a taxonomically diverse range of coral-reef fishes (i.e. Acanthuridae, Apogonidae, Balistidae, Chaetodontidae, and Pomacentridae), we consider A. triostegus a representative teleost model for examining the effects of anthropogenic stressors on fish recruitment via their impacts on metamorphic processes.
Sampling strategy	Sample sizes depended on our success to catch wild larval fish, at night, underwater, using a crest net, at a remote reef location. Sampling was therefore challenging and took several years, in order to obtain a sufficient number of biological and technical replicates for each experiment, based on what is usually done in the literature (see Holzer et al., 2017 - eLife 6:e27595).
Data collection	M.B., W.E.F., I.M. and L.F. collected, investigated and analyzed the data under the guidance of V.L. and D.L
Timing and spatial scale	This study was conducted from February 2015 to June 2018, as it required numerous larval fish samples that were collected nightly in a remote region of the world (French Polynesia) and at a remote reef location (Tema'e at Moorea Island). Most sampling were done around new moon periods, as this species colonize the reef at these periods.
Data exclusions	In the flume experiment, fish that did not swim actively during the first acclimation period were removed from the analysis (n = 3) to prevent side preference bias. Fish that spent more than 50% of the test time in the drain area (i.e. where the two water sources mix)

were also removed from the analysis (n = 4) as we considered that they did not show any particular preference or avoidance for any of the two water sources.

In the visual stimulus experiment, fish that remained immobile and spent more than 50% of the test time in the 'no choice' area were removed from the analysis (n = 11) as we considered that they did not show any particular preference or avoidance for any of the two visual stimuli.

These data exclusion criteria were pre-established.

Reproducibility	Experiments were conducted based on similar experimental approaches already published in the literature (same experimental set up but different problematic), to ensure the reproducibility of experimental findings.<br>All attempts to repeat the experiments were successful (between n = 2 and n= 5 independant replicates for sensory development and behavior analyses, between n = 5 and n = 7 independant replicates for survival experiments, and between n = 2 and n = 4 independant replicates for TH levels analyses).
Randomization	For the predation experiment, color tags attributed to each fish group were randomly switched between each replicate to ensure no predation bias based on tag color.<br>For all experiments, fish were randomly allocated into groups right after sampling.
Blinding	Blinding was not relevant to our study as fish were associated to one treatment immediately after their capture.

Did the study involve field work? ☒ Yes ☐ No

Field work, collection and transport

Field conditions	Larval fish were sampled at night, using a crest net. Sampling effort was particularly focused on periods before and after the new moon, as settlement of larval Acanthurus triostegus principally occurs at these periods (Besson et al., 2017 - PlosOne 12(6): e0178795).
Location	This study was conducted at Moorea Island, French Polynesia (S17°32'16.4589", W149°49'48.3018").
Access and import/export	This study did not involve endangered or protected species and was performed on larval Convict Surgeonfish, Acanthurus triostegus, who exhibit 90% mortality within two days following their entry in the reef. The ecological impact of our sampling towards this species is therefore minimal.
Disturbance	No disturbance was caused by this study.

Reporting for specific materials, systems and methods

We require information from authors about some types of materials, experimental systems and methods used in many studies. Here, indicate whether each material, system or method listed is relevant to your study. If you are not sure if a list item applies to your research, read the appropriate section before selecting a response.

Materials & experimental systems

n/a	Involved in the study
☒	☐ Antibodies
☒	☐ Eukaryotic cell lines
☒	☐ Palaeontology
☐	☒ Animals and other organisms
☒	☐ Human research participants
☒	☐ Clinical data

Methods

n/a	Involved in the study
☒	☐ ChIP-seq
☒	☐ Flow cytometry
☒	☐ MRI-based neuroimaging

Animals and other organisms

Policy information about studies involving animals; ARRIVE guidelines recommended for reporting animal research

Laboratory animals	The study did not involve laboratory animals
Wild animals	Settlement-stage A. triostegus larvae were collected nightly using crest nets on the fringing reef at Tema'e, on the north-east coast of Moorea island (S17°29'49.7362", W149°45'13.899"). Larvae were then transferred to either in situ cages (for thyroid hormone signaling experiments) where they remained for up to 8 days, or to aquaria at the CRIOBE Marine Research Station (for increased temperature and chlorpyrifos exposure experiments) where they remained for up to 5 days. All fish were euthanized in 4°C MS222 at the end of each experiment.
Field-collected samples	For thyroid hormone signaling experiment, following collection and subsequent treatment, larvae were transferred to a nursery area on the north coast of the island (S17°29'26.5378", W149°53'29.2252") where they were raised in in situ cages (cylindrical cages, diameter: 30 cm, height: 50 cm, 15 fish per cage). This allowed them to develop in in situ conditions5. As A. triostegus

feeds on algal turf following settlement5,24, cages were stocked with a supply of turf-covered coral rubble that was replaced daily, ensuring both shelter and constant food availability. Fish were subsequently sampled on d2, d5, and d8, post-collection to examine sensory organ maturation, behavior (response to sensory cues), and survival (predation test).

For increased temperature and chlorpyrifos (CPF) exposures, following collection, fish were maintained in groups of 10 individuals at the CRIOBE Marine Research Station. Each group was held in a 30Lx20Wx20H cm aquaria containing 12 L of filtered (1 µm filter) seawater. All tanks were subject to a 12:12 hour light-dark cycle (06:00 – 18:00 light period) and oxygenated with an air stone. For increased temperature treatments, seawater was in an open system, and water temperature was maintained at either 28.5°C, 30.0°C or 31.5°C depending on the treatment condition. Aquaria used for CPF exposure treatments and associated controls were in a close system and not equipped with coral rubble to prevent potential interaction with the pesticide. Water was replaced each day to ensure the maintenance of CPF concentrations.

Ethics oversight

This study did not involve endangered or protected species and was carried out in accordance with the guidelines of the French Polynesia code for animal ethics and scientific research (https://www.service-public.pf/diren/partager/code/). All protocols and experiments were approved by the CRIOBE-IRCP animal ethics committee (DL-20150214).

Note that full information on the approval of the study protocol must also be provided in the manuscript.
